# IR813-Induced Photothermal Therapy: Leveraging Immunogenic Cell Death for Cancer Treatment

**DOI:** 10.3390/pharmaceutics17020166

**Published:** 2025-01-26

**Authors:** Guangwei Jiang, Rong Huang, Min Qian, Wenjuan Hu, Rongqin Huang

**Affiliations:** School of Pharmacy, Key Laboratory of Smart Drug Delivery (Ministry of Education), Minhang Hospital, Fudan University, Shanghai 201203, China

**Keywords:** photothermal therapy, triple-negative breast cancer, immunogenic cell death, antitumor immunity

## Abstract

**Background**: Photothermal therapy has the potential to enhance the precision and safety of oncological treatments. However, applicable photothermal agents associated with its photothermal activated immunogenic cell death remain exploiting. **Methods**: This study evaluates the effectiveness of IR813, a photothermal agent, combined with near-infrared (NIR) light for cancer treatment. In vitro, 4T1 cancer cells were treated with IR813 (5 μg/mL) and exposed to NIR irradiation (1 W/cm^2^) for 5 min. In vivo, after the tumor-bearing mice administered with IR813 (1 mg/kg) and exposed to NIR irradiation (1 W/cm^2^) for 10 min, the tumor volume, survival and immune activation were evaluated. **Results**: IR813 significantly increased the cytotoxicity of 4T1 cancer cells following near-infrared irradiation, resulting in the release of damage-associated molecular patterns and immunogenic cell death. Specifically, the cell viability was reduced to 5% compared to the control group. In vivo, irradiating the accumulation of IR813 at the tumor site had the potential to mediate substantial photothermal tumor suppression, improved mouse survival, and reduced metastasis, with minimal adverse reactions. Furthermore, the immune responses stimulated by IR813-induced photothermal therapy were evidenced by increased mature dendritic cell and cytotoxic T lymphocyte counts and a decrease in regulatory T cells in the spleen, tumor, and lymph nodes. **Conclusions**: These findings suggest that IR813-induced photothermal therapy is a promising approach for enhancing immunotherapy, directly inhibiting tumors while boosting systemic anti-cancer immunity.

## 1. Introduction

In the ongoing effort to overcome key challenges in cancer therapy, such as tumor recurrence [1], metastasis [2], and side effects [3], the scientific community [4] continues to explore innovative therapeutic strategies to enhance efficacy and minimize adverse reactions. Photothermal therapy, a promising new treatment method, uses near-infrared (NIR) light converted into heat by photothermal agents to selectively kill tumor cells [5,6,7]. Photothermal therapy offers targeted treatment with potentially lower systemic toxicity [8,9]. In recent years, there has been a notable emergence of molecules with near-infrared absorption and photothermal effects, which have become widely utilized in the activation of immunity for the purpose of photoimmunotherapy in the treatment of tumors [10,11,12]. As a high-end NIR fluorescence probe, IR813 has clearly demonstrated its key role in biomedical imaging, primarily because of its deep tissue penetration and minimal background interference. IR813, when doped into silica nanoparticles, has the properties of high brightness, photostability, and non-toxicity. These enable efficient hepatobiliary excretion and accurate lymph node mapping. Similarly, the use of IR813 in the synthesis of red-emitting carbon dots results in far-red emission and has potential for solvent polarity sensing and applications in in vivo imaging and drug delivery [13,14,15]. As a kind of cyanine derivatives, IR813 has demonstrated its potential as a highly efficient infrared absorber, capable of converting absorbed light energy into thermal energy [16]. This unique photothermal conversion property not only strengthens its position in high-resolution biomedical imaging but also opens new avenues for a wide range of photothermal therapy applications. It can reach the tumor through blood circulation and trigger cell death upon NIR exposure. Beyond its direct effects, the immunogenic cell death induced by IR813 with NIR light is of particular interest [17,18,19,20]. This process may activate the immune system to detect and destroy lingering cancer cells.

This study aims to determine the potential of IR813 in combination with NIR to induce immunogenic cell death in tumor cells. We administered 5 μg/mL of IR813 to 4T1 breast cancer cells and found that under NIR irradiation, significant cell death occurred within 3 h. Immunofluorescence and ELISA kits were used to detect the release of immunogenic substances such as ATP, calreticulin (CRT), and high-mobility group box 1 (HMGB1). In vivo, we analyzed the proportion of mature DCs in sentinel lymph nodes to confirm tumor antigen exposure; we analyzed changes in cytotoxic T lymphocytes (CTLs) and inhibitory Treg cells in splenic cells to determine immune activation in mice; and we detected the infiltration of CTLs and a reduction in Tregs at the tumor site, indicating an improvement in the tumor immune microenvironment. Finally, by analyzing tumor growth and survival in tumor-bearing mice, we confirmed the potential therapeutic effects of photothermal-induced ICD on tumors. Through detailed in vitro and in vivo experiments, we aimed to investigate the mechanisms by which IR813 and NIR treatment release damage-associated molecular patterns (DAMPs) that lead to the release of calreticulin (CRT), HMGB1, and ATP and, shortly thereafter, induce an immune response [21,22]. Our findings cover immune reactions in key sites like the spleen, tumor tissue, and lymph nodes, providing insights into the extensive immunomodulatory effects of this therapy.

**Figure 1:**
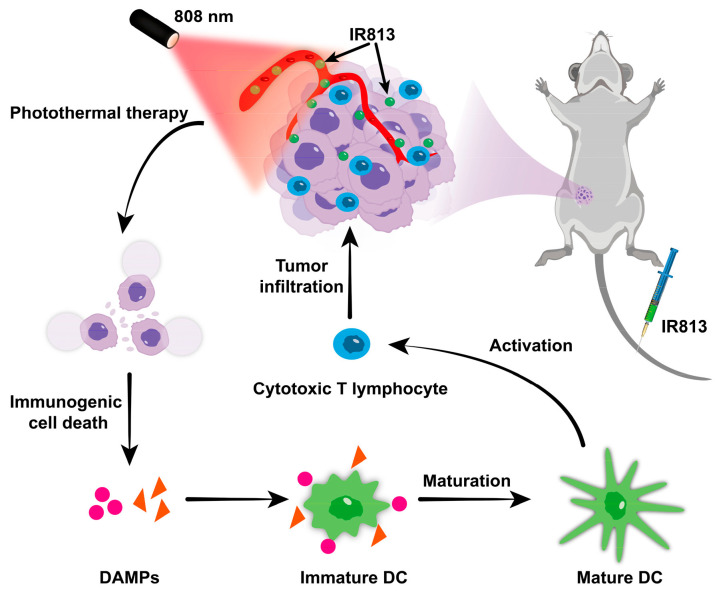
Illustration of photothermal antitumor process using IR813.

As illustrated in Figure 1, this research promises to deepen our understanding of how IR813 functions as a photothermal agent and explores its ability to enhance the immunogenic cell death pathway and support the immune system in fighting tumor progression. This could lead to innovative, integrated cancer treatment strategies that combine photothermal treatments and immunotherapy, pushing forward the development of personalized and effective cancer care.

## 2. Materials and Methods

### 2.1. Materials and Reagents

The supplier of IR-813 p-toluenesulfonate (IR813) was Aladdin (Shanghai, China). The supplier of the ATP bioluminescence assay kit was Beyotime Biotechnology (Shanghai, China). Meilunbio Co. (Dalian, China) was the supplier of 4′,6-Diamidino-2-phenylindole (DAPI), the Annexin V-FITC/PI apoptosis detection kit, the Cell Counting Kit-8, the reactive oxygen assay kit, and the LIVE/DEAD cell imaging kits. Tumor Tissue Digestion Solution was provided by bioGenous (Suzhou, China). Penicillin/streptomycin (PS) and RPMI-1640 media were purchased from Thermo Fisher (Waltham, MA, USA).

### 2.2. Antibodies

Anti-calreticulin (ab92516) and anti-HMGB1 (ab79823) were bought from Abcam. FITC goat anti-rabbit IgG (H + L) (AS011) was purchased from ABclonal (Wuhan, China). The fluorochrome-labeled anti-mouse antibodies used for flow cytometry measurement were bought from Elabscience (Wuhan, China), which included FITC-CD11c (E-AB-F0991C), APC-CD80 (E-AB-F0992E), PE-CD86 (E-AB-F0994D), PE/Cyanine5-CD3 (E-AB-F1013G), FITC-CD4 (E-AB-F1097C), APC-CD8a (E-AB-F1104E), PE-CD8a (E-AB-F1104D), and PE-Foxp3 (E-AB-F1238D). APC-Granzyme B (372204) was bought from Biolegend (San Diego, CA, USA).

### 2.3. Cells and Animals

The Institute of Biochemistry and Cell Biology (IBCB, Shanghai, China) provided the 4T1 cells, which were mouse models of breast cancer. The culture media used were 1640 for 4T1 and MCF-7 cells and DMEM for GL261 and U87 cells added with 10% FBS, 1% penicillin, and 1% streptomycin. The cells were placed at 37 °C with 5% CO_2_ in a humidified incubator. Lingchang Animal Technology Co. (Shanghai, China) provided the BALB/c mice (6–8 weeks, female). The Fudan University Animal Ethics Committee approved the protocols that were followed when conducting the experiments on the BALB/c mice (Approval No. 2021-03-YJ-HRQ-07).

### 2.4. The Spectral Properties of IR813

The spectral properties of IR813 were analyzed using DMSO at a concentration not exceeding 0.5%. Solutions of IR813 at concentrations from 5 to 100 μg/mL were scanned using a UV-visible spectrophotometer (UV-2401PC). Absorbance values were measured at 813 nanometers to construct a calibration curve and perform quantitative analysis. Fluorescence scans were performed using either a fluorescence spectrophotometer (Edinburgh FS5; excitation: 765 nm) or a small animal in vivo imaging system (Caliper; excitation: 765 nm; emission: 820 nm) on solutions of IR813 at concentrations of 1, 2, 3, 4, and 5 μg/mL to determine their fluorescent properties.

### 2.5. Photothermal Effect of IR813

To guarantee the sufficient solubility of IR813, we used dimethyl sulfoxide (DMSO) as a cosolvent, with its concentration strictly kept below 0.5%. The experimental procedure consisted of preparing 0.5 mL aliquots of IR813 solutions at different concentrations, specifically 0, 5, 10, 20, 30, 40, and 50 μg/mL, in a set of 1.5 mL centrifuge tubes. Subsequently, the prepared solutions underwent irradiation with an 808 nm laser with a power intensity of 1 W/cm^2^ for 3 min. Finally, the temperature variations during the irradiation process were recorded in real time using an infrared thermographic camera (FLIR; emissivity: 0.95; reflected temperature: 25 °C; humidity: 50%; distance: 20 cm; thermal sensitivity: ≤0.001 °C; frame rate: 1 Hz).

### 2.6. Cytotoxicity of IR813 + NIR

After seeding 4T1 cells (1 × 10^5^ cells per milliliter) in 6-well plates (2 mL) and 96-well plates (0.1 mL) overnight, the cells in the 6-well plates were treated with IR813 (5 μg/mL) for different lengths of time. Then, the cells were detached from the 6-well plates and analyzed with flow cytometry. The cells in the 96-well plates were given treatment with IR813 + NIR or ICG + NIR, which involved incubating the cells with different concentrations of IR813 or ICG for 2 h, washing the cells three times in PBS, and then irradiating them for 5 min with an 808 nm laser (1 W/cm^2^). Through the Cell Counting Kit-8 and the Cytotoxic LDH Assay Kit-WST, the viability of the cells was assessed after a 3 h, 24 h, and 48 h incubation period. Additionally, the cells were examined using Annexin V-FITC/PI and a LIVE/DEAD cell imaging kit after a 3 h incubation period.

After seeding U87, MCF-7, and GL261 cells (1 × 10^5^ cells per milliliter) in 96-well plates (0.1 mL) overnight, the cells were exposed to IR813 + NIR (5 μg/mL, 808 nm, 5 min, 1 W/cm^2^), and the morphology of the cells was obtained using brightfield illumination and an inverted fluorescence microscope. Through the Cell Counting Kit-8 and the Cytotoxic LDH Assay Kit-WST, the viability of the cells was assessed after a 3 h incubation period.

### 2.7. Evaluation of ROS

After being planted (1 × 10^5^ cells per milliliter, 0.5 mL) in glass-bottom 35 mm dishes, 4T1 cells were cultured for a whole night. IR813 + NIR (5 μg/mL, 2 h incubation, 808 nm, 5 min, 1 W/cm^2^) was then used to treat the cells. The medium was then taken out, and DCFH-DA solution was added. This was left to incubate for 30 min. Lastly, the cells were examined under CLSM after being cleaned with PBS.

### 2.8. Immunofluorescence

Following the seeding of 4T1 cells (1 × 10^5^ cells per milliliter, 0.5 mL) in glass-bottom 35 mm dishes, the cells were cultured for 24 h. Following this, the cells underwent IR813 + NIR (5 μg/mL, 2 h incubation, 808 nm, 5 min, 1 W/cm^2^) therapy. After an additional three hours of incubation, the cells were fixed with 4% formaldehyde to identify HMGB1. After permeabilizing the cells for 5 min with permeabilization buffer, the cells were incubated for 1 h with 1% BSA. Following the addition of anti-HMGB1, the cells were incubated at 4 °C for one night. Subsequently, they were stained with FITC goat anti-rabbit IgG (H + L) in compliance with the manual. DAPI was utilized to label nuclear DNA. In order to detect CRT, the cells were first blocked for one hour with 1% BSA. They were then treated with anti-calreticulin for an additional night at 4 °C and finally at room temperature. Nuclear DNA was labeled with DAPI. The cells were incubated with 1% BSA for 1 h in order to detect CRT. After that, they were treated with anti-calreticulin for an additional night at 4 °C. Subsequently, they were stained with FITC goat anti-rabbit IgG (H + L) in compliance with the manual. With DAPI, nuclear DNA was identified. The cells were then examined under CLSM after being washed with PBS.

### 2.9. ATP Release Detection

After being seeded in 96-well plates (1 × 10^5^ cells per milliliter, 0.1 mL), 4T1 cells were planted for 24 h. Following this, the cells were treated with IR813 + NIR (5 μg/mL, 2 h incubation, 808 nm, 5 min, 1 W/cm^2^) and incubated for another 3 h at 37 °C. Following a 5 min centrifugation at 400× *g*, 20 µL of the supernatant was collected to detect the release of ATP according to the instruction booklet. A luminometer was used to measure the chemiluminescence.

### 2.10. In Vivo Antitumor Study

Female Balb/c mice (6–8 weeks) were given subcutaneous injections of 100 µL of 4T1 cells (1 × 10^6^ cells per mouse) in the breast fat pad to generate an animal model of an orthotopic tumor. At approximately 100 mm^3^, the tumor volume was measured in the mice, and they were randomized to undergo treatment with IR813 + NIR (1 mg/kg) or the control (*n* = 12). After administration in the tail vein, the mice in the IR813 + NIR group were monitored by a small animal in vivo imaging system (Caliper) at a designed point (excitation: 710 nm; emission: 820 nm). As for the IR813 + NIR group, the tumor was radiated with an 808 nm laser for 10 min (1 W/cm^2^) after receiving an intravenous injection of IR813 for 2 h and the thermal imaging results were acquired with FLIR (emissivity: 0.95; reflected temperature: 25 °C; humidity: 50%; distance: 20 cm; thermal sensitivity: ≤0.001 °C; frame rate: 1 Hz). Every 2 days, measurements were taken of the mice’s body tumor size and weight. The formula V = W^2^L/2 was used to determine the tumor size, where W and L represent the smallest and longest diameters, respectively. To assess immune activation, three mice were randomly selected and the tumors, spleen, and lymph nodes were collected four days after treatment. Cut into small pieces, the collected tumors were dipped into Tumor Tissue Digestion Solution to form a single-cell suspension solution. To obtain a suspension of tumor cells, this solution was squeezed gently through a 70 μm nylon membrane after being incubated for 45 min at 37 °C. To produce single-cell suspensions, the removed spleen and lymph nodes were also passed through a 70 μm nylon membrane. The individual cells were stained using fluorescently tagged antibodies, following the manufacturer’s instructions. Anti-CD3, anti-CD4, and anti-Foxp3 antibodies were used to stain lymphocytes in order to analyze regulatory T cells (Tregs; CD3^+^CD4^+^Foxp3^+^), following the manufacturer’s instructions. CD3^+^(CD8^+^/CD4^+^) cells were stained with anti-CD3, anti-CD4, and anti-CD8 antibodies in order to analyze immunological activity. To assess cytotoxic T cells (CD3^+^CD8^+^Granzyme B^+^), anti-CD3, anti-CD8, and anti-granzyme B antibodies were used to stain the lymphocytes. On day twenty-one, three mice were randomly selected and sacrificed, and their organs were taken for histological analysis. This analysis made use of hematoxylin and eosin staining.

### 2.11. Statistical Analysis

The mean plus or minus the standard deviation is displayed for every data point. Data analysis for two groups was evaluated using an independent sample *t*-test using GraphPad Prism software 8, and for multiple groups, one-way analysis of variance (ANOVA) was adopted. The following are the *p*-values for the data points. To denote statistical significance, the following symbols are used: * *p* < 0.05; ** *p* < 0.01; *** *p* < 0.001; and **** *p* < 0.0001.

## 3. Results

### 3.1. Characterization

**Figure 2:**
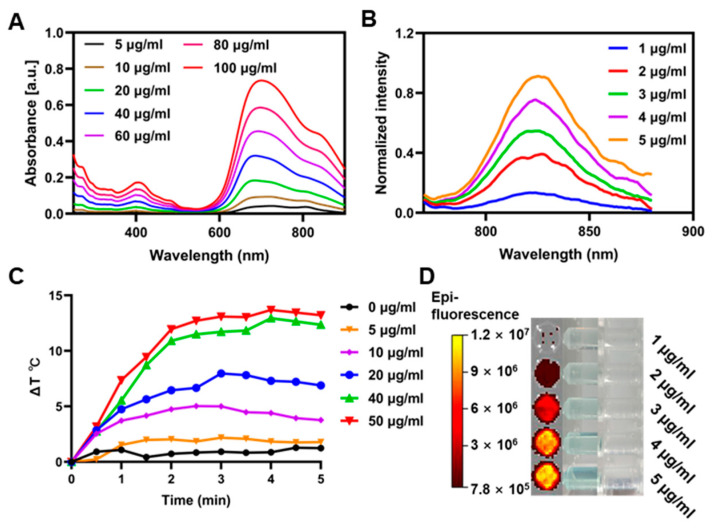
Characterization of IR813. (**A**) UV-vis-NIR spectra of IR813 at various concentrations. (**B**) Fluorescence spectra of IR813 at different concentrations. Excitation wavelength = 765 nm. (**C**) Photothermal conversion curves for different concentrations (0–50 µg/mL) of IR813 with 808 nm photoirradiation at 1 W/cm^2^. (**D**) Fluorescence image and optical images of IR813 solution at different concentrations (excitation: 710 nm; emission: 820 nm).

IR813 is a hydrophobic molecule. To solubilize it, we used a small amount of DMSO, enhancing its distinctive absorption characteristics in the NIR region, particularly the pronounced peak at 813 nm (Figure 2A). The absorption strength showed a strong linear correlation with concentrations ranging from 5 to 100 µg/mL, proving highly accurate as a quantitative probe (Figure S1). Additionally, IR813 demonstrated exceptional long-wavelength fluorescence properties, emitting a robust spectral signal at approximately 820 nm following excitation with 740 nm light. The fluorescence intensity at this wavelength maintained a direct proportionality with concentrations between 1 and 5 µg/mL, underscoring its potential for in vivo fluorescence imaging (Figure 2B,D). Furthermore, IR813 showed excellent photothermal conversion performance. Under laser irradiation at a power density of 1 W/cm^2^, 40 µg/mL IR813 solution could achieve a temperature increase of approximately 10 degrees Celsius (Figure 2C), with this photothermal heating being concentration-dependent. These results confirm IR813′s significant potential as an agent for both fluorescence imaging and photothermal conversion in biomedical applications.

### 3.2. Immunogenic Cell Death

**Figure 3:**
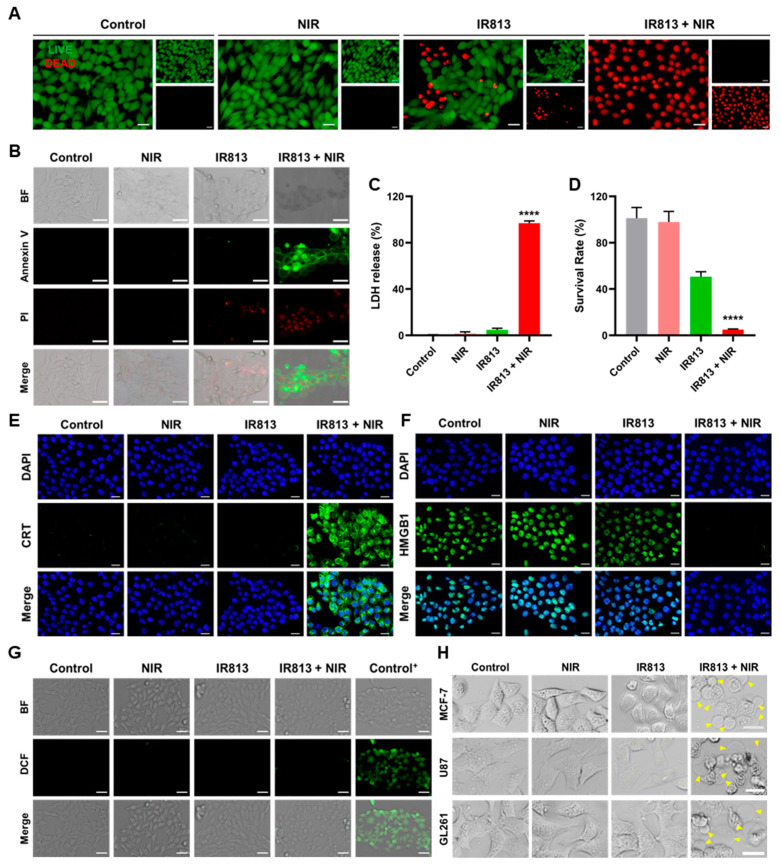
In vitro cancer cell killing effects. (**A**) CLSM pictures of 4T1 cells after distinct treatments that were labeled using a live/dead cell imaging kit. (**B**) CLSM pictures of 4T1 cells after various treatments that were stained using an apoptosis detection kit called Annexin V-FITC/PI. (**C**) LDH release from 4T1 cells processed with different formulations after 3 h. (**D**) The survival rate of 4T1 cells treated with varied formulations after 3 h. (**E**) Images of CRT-immunofluorescence-labeled 4T1 cells taken with a confocal microscope under different conditions. (**F**) Images of HMGB1-immunofluorescence-labeled 4T1 cells taken with a confocal microscope under various conditions. (**G**) Images of 4T1 cells stained with DCFH under dark conditions following treatment with various formulations. The ROS assay kit’s positive control, Rosup, was utilized to process the Control^+^ group. (**H**) Typical bright-field images of GL261, U87, and MCF-7 cells following treatment. The yellow triangles pointed to cells with membrane bubbling. All images are scaled to 20 μm. The data presented in each set are represented by the mean ± standard deviation (SD), with a sample size of *n* = 3. **** *p* < 0.0001.

After 2 h, 4T1 tumor cells were first exposed to an 808 nm NIR laser for 5 min (1 W/cm^2^), which was sufficient for the cells to take up the maximum value of IR813 (Figure S2). The results revealed significant cell damage, as indicated by live/dead cell imaging assays, and most of the treated cells stained positive for propidium iodide (PI) (Figure 3A), highlighting the lethal efficacy of IR813 + NIR in 4T1 cells. Further investigations using LDH release and CCK-8 cell viability assays confirmed a dramatic reduction in cell viability to less than 5% post-treatment (Figure 3C,D and Figure S3), robustly supporting the superior antitumor activity of IR813 + NIR, which was more effective than ICG + NIR in the same conditions (Figure S4). Detailed analysis showed that treated tumor cells not only displayed typical signs of cell death but also exhibited cytoplasmic vacuolation and a significant increase in membrane bubbling (Figure 3B), characteristics of pyroptosis [23,24]. This suggests that the treatment may have induced immunogenic cell death. Following IR813 + NIR therapy, increased CRT exposure on the surface of the endoplasmic reticulum and release of HMGB1 from nuclei into the extracellular environment were demonstrated by immunofluorescence staining (Figure 3E,F). Additionally, there was a massive ATP release (Figure S2) and elevated levels of DAMPs (Figure 3E,F and Figure S5), indicating a potential to activate immune responses. Notably, no significant ROS generation was detected in the cells post-treatment (Figure 3G), suggesting that the cell death mechanism may not have relied on conventional ROS-mediated pathways. Overall, the combination of IR813 with NIR irradiation consistently displayed remarkable cytotoxicity and features of pyroptosis across various tumor cell models, including 4T1 (Figure 3H and Figure S6), further underscoring its potential in antitumor therapy. In vitro experiments demonstrated the potent cytotoxic effects of IR813 combined with NIR light irradiation.

### 3.3. In Vivo Antitumor Effects

**Figure 4:**
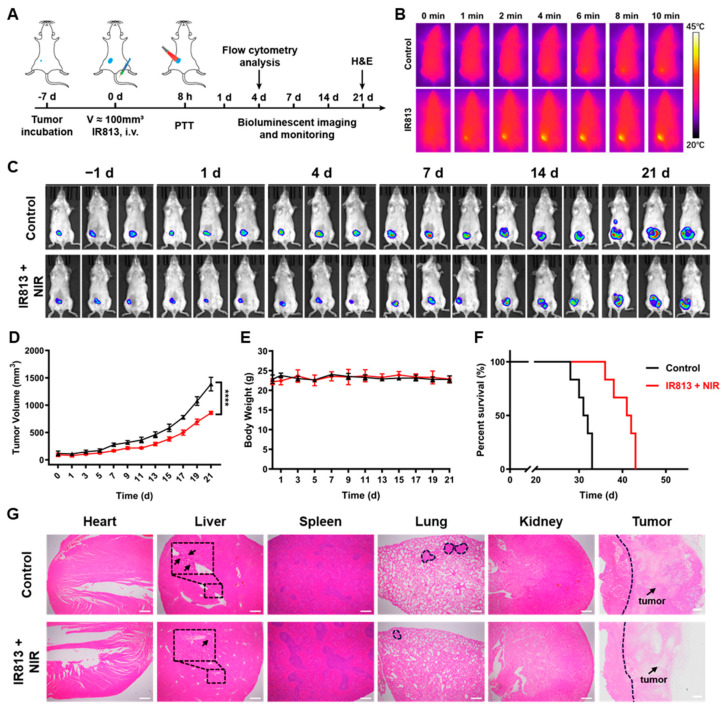
In vivo antitumor effects. (**A**) Treatment schedule for BALB/c mice with 4T1-luci tumor. (**B**) Photothermal images of tumor-bearing mice being administered PBS and IR813 at different times (808 nm, 1 W/cm^2^ NIR irradiation for 10 min at each site). (**C**) Bioluminescent images of mice following various treatments. (**D**) Tumor volume, (**E**) body weight, and (**F**) survival curves for mice following distinct treatments (*n* = 6). (**G**) H&E images of major organs and tumors in various groups. The blank arrows pointed to tumors. Scale bar = 500 μm.

In the in vivo antitumor experiments, a significant temperature increase was observed within the tumor region after the intravenous administration of the NIR dye IR813 at a dosage of 1 mg/kg for 2 h, when IR813 at the tumor site reached its maximum (Figure S7), and when exposure to NIR light was at an intensity of 1 W/cm^2^ for 10 min. Photothermal imaging showed that the temperature increase matched the tumor’s contour rather than forming a uniform circular spot, suggesting the selective accumulation of IR813 in tumor tissues and its potent photothermal conversion properties in vivo (Figure 4B and Figure S8). Further studies confirmed the inhibitory effect of IR813 + NIR on tumor growth. Bioluminescence imaging monitored tumor progression in tumor-bearing mice (Figure 4C), and continuous measurements of tumor volume changes demonstrated that the treatment effectively suppressed tumor proliferation (Figure 4D) and prolonged survival times in these mice (Figure 4F). Using hematoxylin and eosin (H&E) staining for the histopathologic evaluation of the lungs, the results showed a potential reduction in metastatic nodules compared to control groups (Figure S9). Throughout the treatment, the body weights of the mice did not change significantly during the treatment period (Figure 4E), and no pathological changes were observed in major organs such as the heart, spleen, and kidneys, indicating that IR813 did not cause significant systemic toxicity under the administered doses and treatment regimens (Figure 4G).

### 3.4. In Vivo Immune Activation

**Figure 5:**
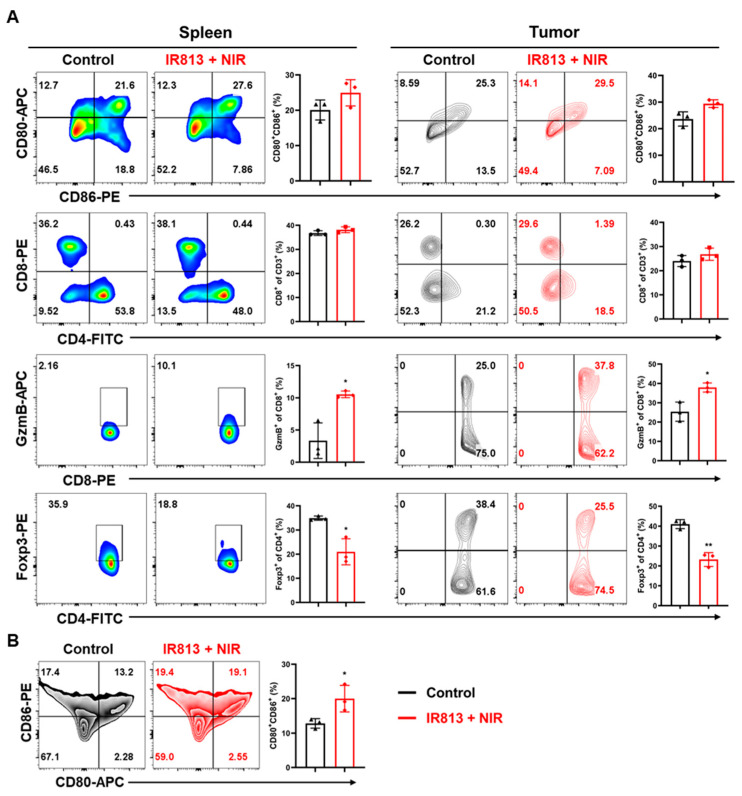
Immune activation effects. (**A**) Typical flow cytometric results and quantification of matured DCs (CD80^+^CD86^+^ DCs) belonging to CD11c^+^ cells, CD8^+^ T cells, cytotoxic T lymphocytes, (CD8^+^Granzyme B^+^) and regulatory T cells (CD4^+^Foxp3^+^) belonging to CD3^+^ cells in spleens and primary tumors. (**B**) Typical flow cytometric results and quantification of matured DCs (CD80^+^CD86^+^ DC cells) belonging to CD11c^+^ cells in inguinal lymph nodes. Mean ± SD (*n* = 3) is employed to present data in all instances. * *p* < 0.05 and ** *p* < 0.01.

Building on previous cellular-level research that confirmed the IR813 + NIR treatment’s ability to trigger significant DAMP release from tumor cells, thereby stimulating immune responses, further in-depth animal model investigations were conducted. We focused on the dynamic changes in immune activation within the spleen, tumor tissue, and tumor-related sentinel lymph nodes, such as the inguinal lymph nodes. The percentage of mature dendritic cells (DCs) in the sentinel lymph nodes increased significantly from 12.8% to 20.0% in the initial studies (Figure 5B), highlighting effective antigen presentation essential for triggering immune responses. Subsequent analysis revealed notable changes in immune cell composition in the spleen. The proportion of mature DCs increased from 21.7% to 26.5%, that of cytotoxic T lymphocytes rose from 3.3% to 10.53%, and that of regulatory T cells decreased from 34.9% to 22.4% (Figure 5A), signaling a significantly activated immune state in mice treated with IR813 and NIR. Direct assessments of intratumoral immune responses corroborated these findings. The percentage of mature DCs within the tumor increased from 23.6% to 29.4%, that of cytotoxic T lymphocytes increased from 25.4% to 37.9%, and that of regulatory T cells decreased dramatically from 41.0% to 23.2% (Figure 5A). These changes indicate that IR813 plus NIR treatment historically enhanced effector T cell infiltration into the tumor core, boosting the immune system’s ability to attack tumor cells and contributing to the inhibition of tumor growth and metastasis.

## 4. Conclusions

This study verifies the therapeutic benefits of IR813, a photothermal agent activated by NIR irradiation, in cancer treatment. In vitro, IR813 + NIR demonstrates potent cytotoxic effects on 4T1 cells by inducing immunogenic cell death and releasing DAMPs, which are crucial for initiating immune responses. In vivo, IR813 + NIR has the ability to suppress tumors and enhance survival rates with minimal toxicity, underscoring its potential for clinical safety. The treatment not only facilitates direct tumor ablation but also prompts a systemic immune response, characterized by increased mature dendritic cells and cytotoxic T lymphocytes and decreased regulatory T cells at key immune sites. The dual actions of photothermal and immune stimulation make IR813 + NIR therapy a promising approach to augment cancer immunotherapy, combining tumor destruction with immune system engagement to curb growth and prevent metastasis. Compared to the commonly used near-infrared photothermal reagent indocyanine green (ICG), IR813 demonstrates rapid and effective cell-killing capabilities at lower concentrations in vitro, and it can quickly expose immunogenic substances. The dual actions of photothermal and immune stimulation make IR813 + NIR therapy a promising approach to augment cancer immunotherapy, combining tumor destruction with immune system engagement to curb growth and prevent metastasis. Future developments could focus on optimizing the delivery methods of IR813, improving targeting specificity, and exploring synergistic combinations with other therapeutic modalities. Additionally, large-scale clinical trials are essential to fully evaluate the safety and efficacy of IR813 in diverse patient populations. These results advocate for the advancement of IR813-based therapies, presenting a new, minimally invasive, and potent option for cancer treatment that leverages the unique properties of IR813 and NIR for a comprehensive antitumor response.

## Data Availability

The original contributions presented in this study are included in the article/Supplementary Material. Further inquiries can be directed to the corresponding author.

## Supplementary Materials

The following supporting information can be downloaded at https://www.mdpi.com/article/10.3390/pharmaceutics17020166/s1. Figure S1. (a) Optical images of IR813 solution at different concentrations. (b) Quantification curve of IR813 through UV-vis-NIR spectra. Figure S2. (a) Representative flow cytometry histogram and (b) corresponding mean fluorescence intensity of IR813 uptake by 4T1 cells. All data are represented as mean ± SD (*n* = 3). **** *p* < 0.0001. Figure S3. (a) LDH release and (b) Survival rate of 4T1 cells treated with IR813 or IR813 + NIR after 3 h, 24 h and 48 h. Figure S4. (a) Survival rate and (c) LDH release of 4T1 cells treated with IR813 or IR813 + NIR after 3 h. (b) Survival rate and (d) LDH release of 4T1 cells treated with ICG or ICG + NIR after 3 h. All data are represented as mean ± SD (*n* = 3). *** *p* < 0.001, **** *p* < 0.0001. Figure S5. ATP secretion level of 4T1 cells under various treatments. All data are presented as mean ± SD (*n* = 3). ** *p* < 0.01. Figure S6. (a) LDH release and (b) Survival rate of GL261, MCF-7 and U87 cells treated with IR813 + NIR after 3 h. All data are represented as mean ± SD (*n* = 3). **** *p* < 0.0001. Figure S7. (a) In vivo fluorescence images and (b) corresponding semi-quantitative graph for visualizing the retention of IR813 at varied time points after administration (Excitation: 710 nm; Emission: 820 nm). All data are represented as mean ± SD (*n* = 3). Figure S8. (a) Temperature changes of tumor-bearing mice treated with IR813 at different time. (808 nm 1 W/cm^2^ NIR irradiation for 10 min at each point). (b) Semi-Quantitative Temperature Change Curves at the Tumor Site. All data are represented as mean ± SD (*n* = 3). Figure S9. H&E images of lungs from different four tumor-bearing mice treated with Control and IR813 + NIR. Bar = 200 μm. Figure S10. Gating strategy of FACS analysis for detecting CD80^+^CD86^+^ cells in the CD11c^+^ cells and for detecting CD8^+^ T cells, cytotoxic T lymphocytes (CD8^+^Granzyme B^+^) and regulatory T cells (CD4^+^Foxp3^+^) in the CD3^+^ cells of the tumor-bearing mice with different treatments.
