# Supplementary material for: IR813-Induced Photothermal Therapy: Leveraging Immunogenic Cell Death for Cancer Treatment
Source: Pharmaceutics. 2025 Jan 26;17(2):166. doi: 10.3390/pharmaceutics17020166 (PMC11859857; doi:10.3390/pharmaceutics17020166)
Supplement: Supplementary file 1 [file pharmaceutics-17-00166-s001.zip › pharmaceutics-3424665-supplementary.pdf]

IR813-Induced Photothermal Therapy: Leveraging Immunogenic Cell Death for Cancer Treatment

*Guangwei Jiang[a], Rong Huang[a], Min Qian[a] and Wenjuan Hu[*a], Rongqin Huang[*a]*

a. School of Pharmacy, Key Laboratory of Smart Drug Delivery (Ministry of Education), Minhang Hospital, Fudan University, Shanghai 201203, China

E-Mail: rqhuang@fudan.edu.cn, wenjuan_h@fudan.edu.cn, Phone/Fax: 86-21-50751769

Supplementary Material

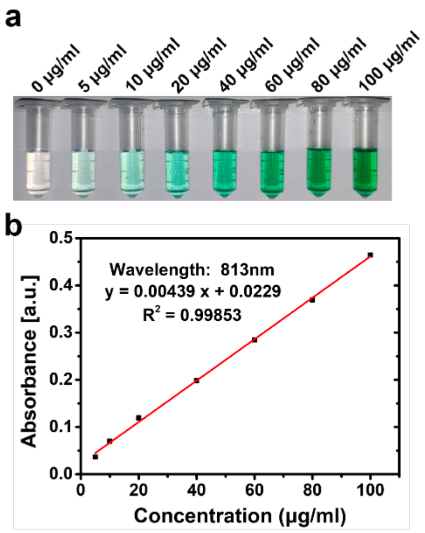

Figure S1. a) Optical images of IR813 solution at different concentrations. b) Quantification curve of IR813 through UV-vis-NIR spectra.

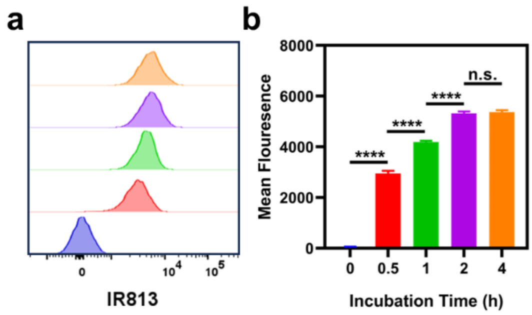

Figure S2. a) Representative flow cytometry histogram and b) corresponding mean fluorescence intensity of IR813 uptake by 4T1 cells. All data are represented as mean ± SD (n = 3). ****p < 0.0001.

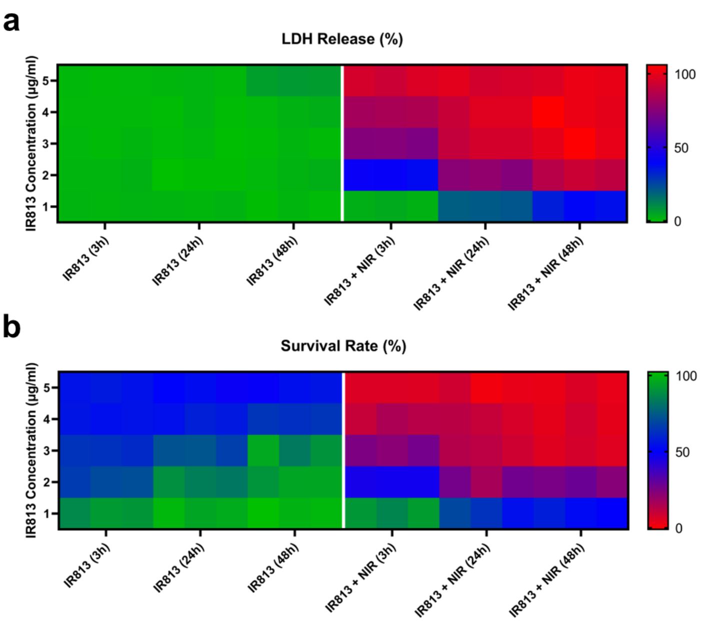

Figure S3. a) LDH release and b) Survival rate of 4T1 cells treated with IR813 or IR813 + NIR after 3 h, 24 h and 48 h.

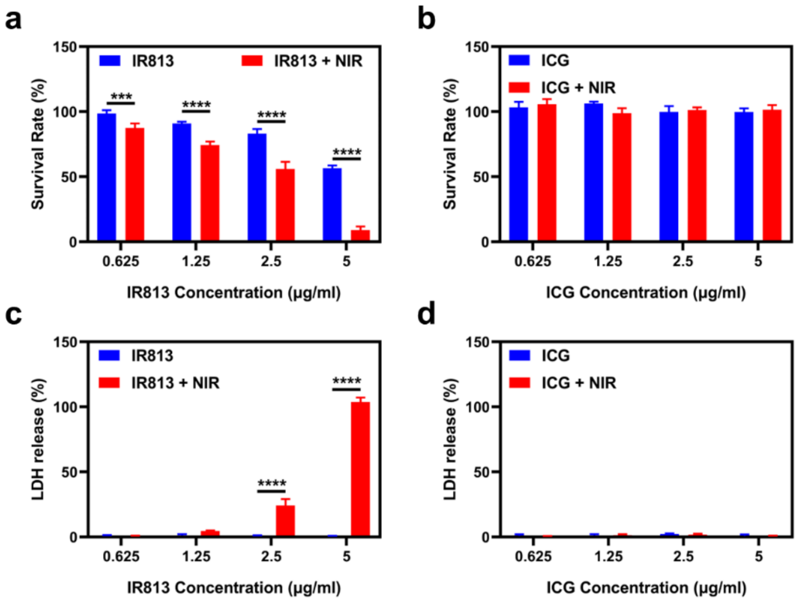

Figure S4. a) Survival rate and c) LDH release of 4T1 cells treated with IR813 or IR813 + NIR after 3 h. b) Survival rate and d) LDH release of 4T1 cells treated with ICG or ICG + NIR after 3 h. All data are represented as mean ± SD (n = 3). ***$p < 0.001$, ****$p < 0.0001$.

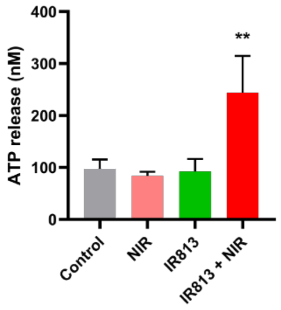

Figure S5. ATP secretion level of 4T1 cells under various treatments. All data are presented as mean ± SD (n = 3). **p < 0.01.

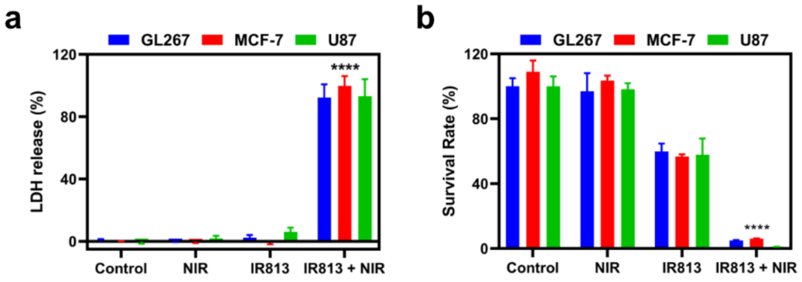

Figure S6. a) LDH release and b) Survival rate of GL261, MCF-7 and U87 cells treated with IR813 + NIR after 3 h. All data are represented as mean ± SD (n = 3). ****p < 0.0001.

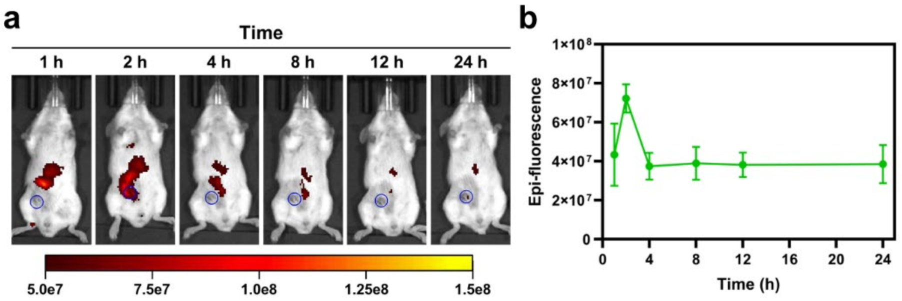

Figure S7. a) In vivo fluorescence images and b) corresponding semi-quantitative graph for visualizing the retention of IR813 at varied time points after administration (Excitation: 710 nm; Emission: 820 nm). All data are represented as mean ± SD (n = 3).

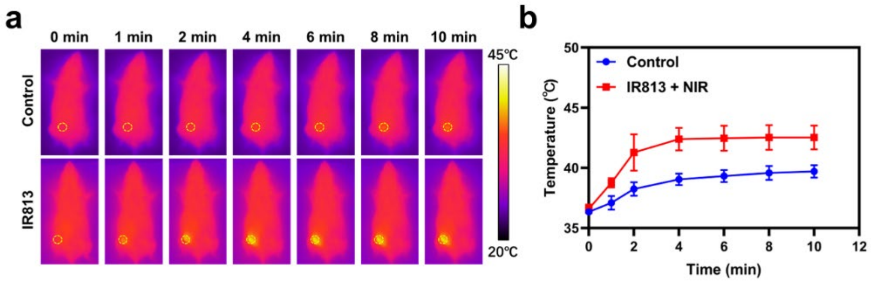

Figure S8. a) Temperature changes of tumor-bearing mice treated with IR813 at different time. (808 nm 1 W/cm^2 NIR irradiation for 10 min at each point). b) Semi-Quantitative Temperature Change Curves at the Tumor Site. All data are represented as mean ± SD (n = 3).

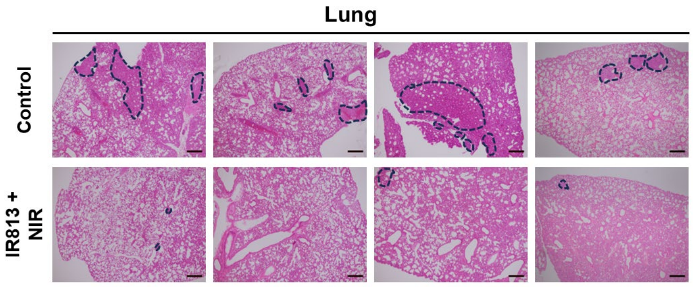

Figure S9. H&E images of lungs from different four tumor-bearing mice treated with Control and IR813 + NIR. Bar = 200 μm.

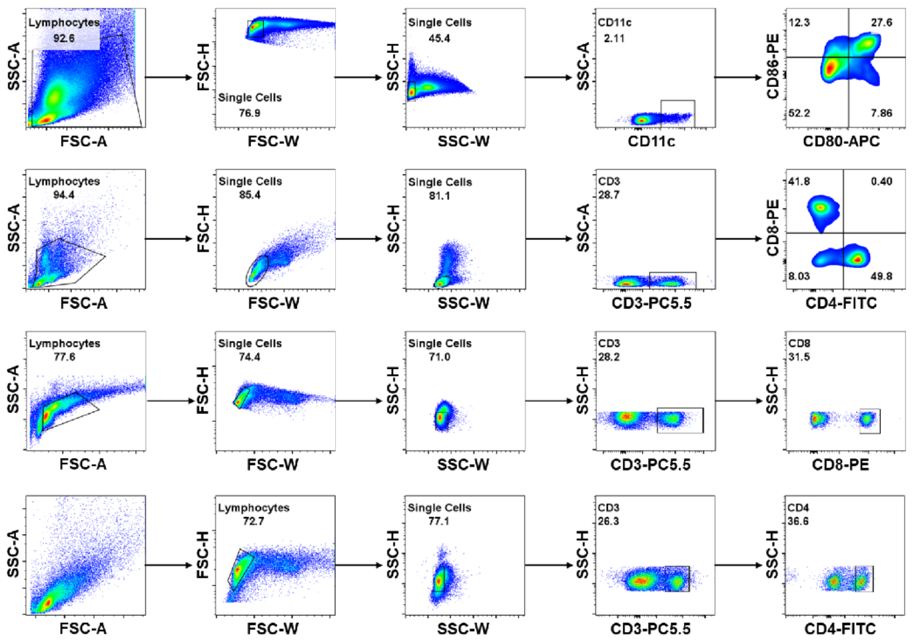

Figure S10. Gating strategy of FACS analysis for detecting CD80$^+$CD86$^+$ cells in the CD11c$^+$ cells and for detecting CD8$^+$ T cells, cytotoxic T lymphocytes (CD8$^+$Granzyme B$^+$) and regulatory T cells (CD4$^+$Foxp3$^+$) in the CD3$^+$ cells of the tumor-bearing mice with different treatments.
